# Brain lesions causing parkinsonism versus seizures map to opposite brain networks

**DOI:** 10.1093/braincomms/fcae196

**Published:** 2024-06-05

**Authors:** Frederic L W V J Schaper, Mae Morton-Dutton, Niels Pacheco-Barrios, Joseph I Turner, William Drew, Sanaz Khosravani, Juho Joutsa, Michael D Fox

**Affiliations:** Center for Brain Circuit Therapeutics, Brigham and Women’s Hospital, Harvard Medical School, Boston, MA 02115, USA; Department of Neurology, Brigham and Women’s Hospital, Harvard Medical School, Boston, MA 02115, USA; Center for Brain Circuit Therapeutics, Brigham and Women’s Hospital, Harvard Medical School, Boston, MA 02115, USA; Department of Neurology, Brigham and Women’s Hospital, Harvard Medical School, Boston, MA 02115, USA; Center for Brain Circuit Therapeutics, Brigham and Women’s Hospital, Harvard Medical School, Boston, MA 02115, USA; Department of Neurosurgery, Brigham and Women’s Hospital, Harvard Medical School, Boston, MA 02115, USA; Center for Brain Circuit Therapeutics, Brigham and Women’s Hospital, Harvard Medical School, Boston, MA 02115, USA; Department of Neurology, Brigham and Women’s Hospital, Harvard Medical School, Boston, MA 02115, USA; Center for Brain Circuit Therapeutics, Brigham and Women’s Hospital, Harvard Medical School, Boston, MA 02115, USA; Department of Neurology, Brigham and Women’s Hospital, Harvard Medical School, Boston, MA 02115, USA; Center for Brain Circuit Therapeutics, Brigham and Women’s Hospital, Harvard Medical School, Boston, MA 02115, USA; Department of Neurology, Brigham and Women’s Hospital, Harvard Medical School, Boston, MA 02115, USA; Turku Brain and Mind Center, Clinical Neurosciences, University of Turku, 20520 Turku, Finland; Turku PET Centre, Neurocenter, Turku University Hospital, 20520 Turku, Finland; Center for Brain Circuit Therapeutics, Brigham and Women’s Hospital, Harvard Medical School, Boston, MA 02115, USA; Department of Neurology, Brigham and Women’s Hospital, Harvard Medical School, Boston, MA 02115, USA; Athinoula A Martinos Center for Biomedical Imaging, Department of Radiology, Massachusetts General Hospital, Harvard Medical School, Charlestown, MA 02129, USA

**Keywords:** Parkinson’s disease, epilepsy, lesion network mapping, human connectome

## Abstract

Recent epidemiological studies propose an association between parkinsonism and seizures, but the direction of this association is unclear. Focal brain lesions causing new-onset parkinsonism versus seizures may provide a unique perspective on the causal relationship between the two symptoms and involved brain networks. We studied lesions causing parkinsonism versus lesions causing seizures and used the human connectome to identify their connected brain networks. Brain networks for parkinsonism and seizures were compared using spatial correlations on a group and individual lesion level. Lesions not associated with either symptom were used as controls. Lesion locations from 29 patients with parkinsonism were connected to a brain network with the opposite spatial topography (spatial *r* = −0.85) compared to 347 patients with lesions causing seizures. A similar inverse relationship was found when comparing the connections that were most specific on a group level (spatial *r* = −0.51) and on an individual lesion level (average spatial *r* = −0.042; *P* < 0.001). The substantia nigra was found to be most positively correlated to the parkinsonism network but most negatively correlated to the seizure network (spatial *r* > 0.8). Brain lesions causing parkinsonism versus seizures map to opposite brain networks, providing neuroanatomical insight into conflicting epidemiological evidence.

## Teaser

Epidemiological studies propose an association between parkinsonism and seizures, but its direction is unclear. Schaper *et al.* report that lesions causing parkinsonism map to the opposite brain network as lesions causing seizures, supporting an inverse relationship and providing neuroanatomical insight into conflicting epidemiological evidence.

## Graphical Abstract

**Graphical Abstract:**
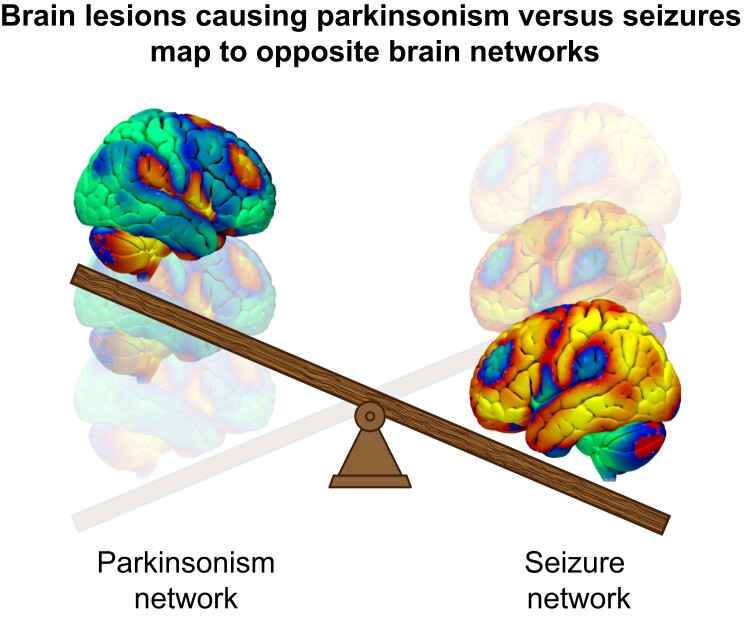

## Introduction

In 1928, Yakovlev^1^ published an influential case series of individuals with epilepsy who developed parkinsonism. Yakovlev proposed that the neuroanatomical damage associated with lifelong seizures may increase the risk of parkinsonism, suggesting shared pathophysiology. In contrast, Yakovlev also noted that in these cases, seizures decreased or even vanished after the onset of parkinsonism, proposing that the neuroanatomical damage associated with parkinsonism may protect against seizures.

Since Yakovlev’s clinical observations, recent epidemiological studies have observed a positive relationship between seizures and parkinsonism; having one diagnosis increases the chances of having the other.^2,3^ However, there is also evidence for an inverse relationship, as seizures can improve parkinsonism^4,5^ and the onset of parkinsonism can improve seizures.^1,4^ Understanding the causal relationship between these two common brain diseases is potentially important for understanding the pathophysiology, prognosis and treatment. However, sorting out this relationship with epidemiology alone is difficult and may become more difficult over time with the increasing availability of treatment. For example, anti-seizure drugs can induce parkinsonism symptoms and may increase the risk of Parkinson’s disease, while neuroleptic drugs can induce both parkinsonism and seizures.^6^

Here, we first collate existing epidemiological data to assess whether the findings favour a positive or negative relationship between parkinsonism and seizures. Next, we investigate this relationship from a unique perspective, examining cases where focal brain lesions cause new-onset parkinsonism versus cases where lesions cause new-onset seizures. If lesions causing these symptoms map to common neuroanatomy, that would suggest shared pathophysiology and support prior observations of a positive relationship. Conversely, if lesions causing these symptoms map to a different neuroanatomy, this could support prior observations of an inverse relationship.

## Materials and methods

This study was carried out in accordance with the Declaration of Helsinki; approved by the institutional review board of the Brigham and Women’s Hospital, Boston, MA; and exempted from obtaining informed consent based on the secondary use of research data.

### Systematic literature review

**Figure 1:**
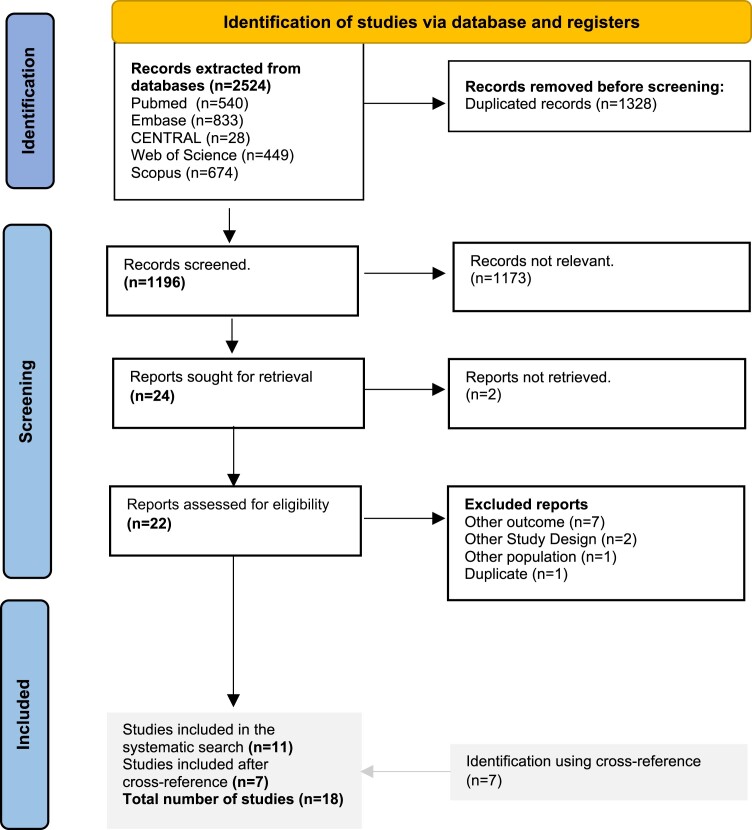
Preferred Reporting Items for Systematic reviews and Meta-Analyses flow diagram for the systematic literature search.

We performed a systematic literature search, following the Preferred Reporting Items for Systematic Reviews and Meta-Analyses guidelines, on PubMed/Medline, Cochrane Central, Embase, Web of Science and Scopus, using the keywords (‘Parkinsonism’, ‘Parkinson’s disease’), (‘epilepsy’, ‘seizures’) and (‘risk factor’) to identify studies reporting on the relationship between parkinsonism and seizures up to 15 February 2024 in English (Fig. 1). A librarian revised the systematic search, and we manually checked the included studies’ reference lists, performing backward and forward citation analyses (e.g. cross-reference), to identify all relevant studies. We included case series, case reports, observational studies (cross-sectional, cohort and case–control studies) and systematic reviews reporting on the association between Parkinson’s disease and seizures. Conference abstracts, books or book chapters were excluded. The selected studies’ design, sample size, primary results, statistical analysis and conclusions are summarized in Table 1. Two observers independently assessed each study and identified whether the results provided qualitative evidence favouring a positive or negative relationship between parkinsonism and seizures. In case of disagreement in the first observation, the observers reached a consensus in a second observation.

**Table 1:** Literature summary on the relationship between parkinsonism and seizures

Author	Year	Study type	*N*	Main result	Favours a:	
					Positive relationship	Negative relationship
Case reports or case series						
Yakovlev	1928	Case series	4	Patients with seizures develop parkinsonism in later life, and the onset of parkinsonism was associated with a decrease in seizures	Both directions	
De Angelis	1984	Case report	1	In a patient with epilepsy, seizures did not change with Parkinson’s disease progression, but motor symptoms temporarily improved after seizures		X
Vercueil	2000	Case report	1	In a patient with seizures, Parkinson’s disease onset was associated with a marked reduction in seizure frequency, and seizures that did develop improved motor symptoms up to 8 days after the seizure		X
Cukiert	2009	Case report	1	In a patient with epilepsy associated with double-cortex syndrome, VNS induced parkinsonian symptoms		X
Silver	2013	Case series	5	Discontinuation of valproic acid for the treatment of seizures, depression or myoclonus improved parkinsonism symptoms		X
Son	2016	Case series	5	Patients with poor seizure control had a more aggressive progression of Parkinson’s disease	X	
Observational studies (cohort, case–control and cross-sectional)						
Bodenmann	2001	Case–control	368	Epilepsy was less frequent in patients with parkinsonism		X
Gaitatzis	2004	Cross-sectional study	1 041 643	Patients with epilepsy have an increased prevalence of Parkinson’s disease [PR (95% CI) = 3.29 (2.48–4.36)], among patients >64 years	X	
Skow	2013	Matched case–control study	50 709	Patients using carbamazepine do not have an increased risk of Parkinson’s disease [OR (95% CI) = 0.93 (0.81–1.08)]	No relationship	
Feddersen	2014	Case–control	3752	Prevalence of epilepsy in Parkinson’s disease is low (2.6%), but SE is more frequent in concomitant Parkinson’s disease and epilepsy compared to patients with epilepsy (*P* < 0.01)	Both directions	
Heilbron	2019	Cross-sectional	53 707	Patients with Parkinson’s disease have an increased odds of epilepsy [OR (95% CI) = 2.80 (1.07–759 184.42)]	X	
Gruntz	2018	Nested case–control	115 429	Patients with Parkinson’s disease have an increased odds of epilepsy [OR (95% CI) = 1.68 (51.43–1.98)]	X	
Jacobs	2020	Case–control (UK Biobank)	502 533	Patients with Parkinson’s disease have an increased odds of epilepsy [OR (95% CI) = 2.65 (1.80–3.89)]	X	
Simonet	2022	Nested case–control (UK Biobank)	1 010 578	Patients with Parkinson’s disease have an increased odds of epilepsy [OR (95% CI) = 2.5 (1.63–3.83)]	X	
Belete	2023	Nested case–control (UK Biobank)	10 031	Patients using AEDs have an increased odds of Parkinson’s disease [OR (95% CI) = 1.80 (1.35–2.40)]		X
Kostev	2023	Case–control	24 950	Patients using AEDs [OR (95 CI) = 1.43 (1.33–1.53)] and patients with epilepsy [OR (95% CI) = 1.91 (1.69–2.15)] have increased odds of Parkinson’s disease	Both directions	
Hwang	2024	Case–control	10 510	Patients with epilepsy have increased risk of Parkinson’s disease [HR (95% CI) = 2.19 (1.55–3.12)]	X	
Meta-analyses						
Takamiya	2021	Meta-analysis	129 (14 studies)	ECT improved motor symptoms in patients with Parkinson’s disease [SMD (95% CI) = 1.181 (0.873–1.489); *P* < 0.001]		X

AEDs, antiepileptic drugs; CI, confidence interval; ECT, electroconvulsive therapy; HR, hazard ratio; *N*, number of patients; OR, odds ratio; PR, prevalence ratio; SE, status epilepticus; SMD, standardized mean difference; VNS, vagal nerve stimulation.

### Lesions

Lesions were obtained from two previously published studies identifying brain networks for parkinsonism^7^ or seizures.^8^ For the parkinsonism network, 29 lesions associated with parkinsonism and 135 lesions not associated with parkinsonism were studied (85 females and 79 males).^7^ For the seizure network, 347 lesions associated with epilepsy and 1126 lesions not associated with epilepsy were studied (920 males and 553 females).^8^

### Lesion network mapping

Lesion network mapping couples the lesion locations causing specific neuropsychiatric symptom with an atlas of human brain connectivity to estimate the network connected to each lesion location in the average human brain.^9^ In this method, the lesion location is commonly derived from the structural brain images of a patient, while the human brain connectivity data is derived from functional MRI data of a large sample of healthy participants (*n* = 1000), also known as a normative connectome.^10^ Here, we use the lesion locations of two previously published studies to compare the lesion networks of parkinsonism^7^ versus epilepsy.^8^

### Parkinsonism versus seizure network

**Figure 2:**
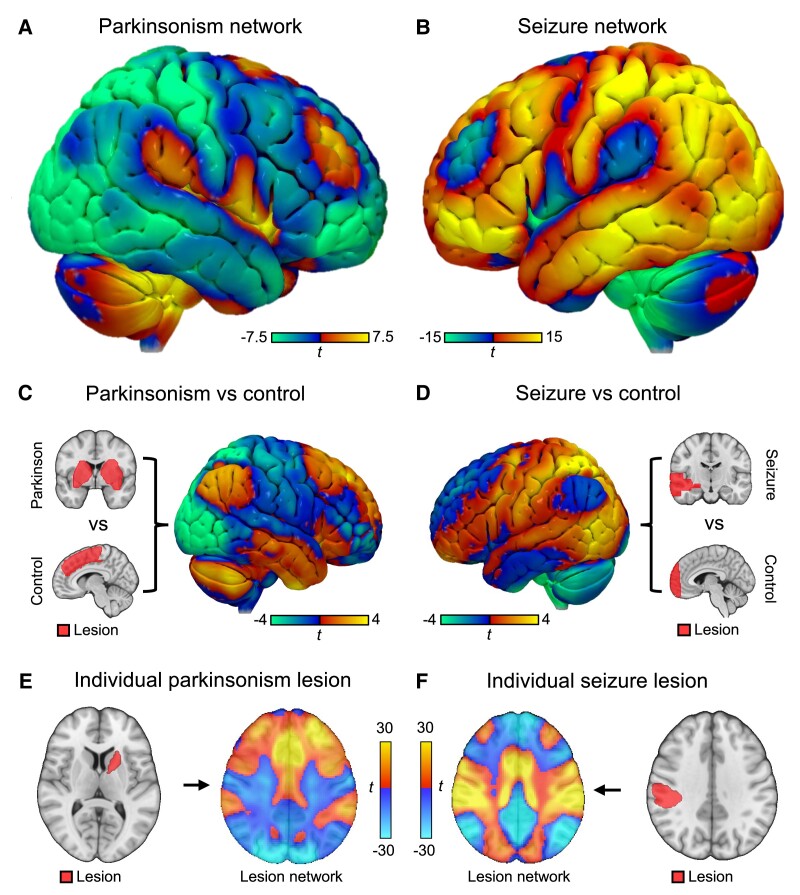
**Brain lesions causing parkinsonism versus seizures map to opposite brain networks.** We previously identified a parkinsonism network (**A**) and a seizure network (**B**) by combining lesions causing new-onset parkinsonism (*n* = 29) or seizure (*n* = 347) with an atlas of normative brain connectivity (the human connectome).^10^ First, we tested the spatial correlation between the published parkinsonism and seizure networks and found they were inversely related (spatial *r* = −0.85). Second, we compared lesions causing parkinsonism (*n* = 29) to control lesions (*n* = 135; **C**) and lesions causing seizures (*n* = 347) to control lesions (*n* = 1126; **D**) and found that these two group-level specificity maps also showed an inverse relationship (spatial *r* = −0.51). Third, we compared each individual parkinsonism (*n* = 29; **E**) and seizure (*n* = 347; **F**) lesions to each other and found a similar inverse relationship [average spatial *r* (95% CI) = −0.042 (−0.0484:−0.0360); one-sample *t*-test *P* < 0.001].

**Figure 3:**
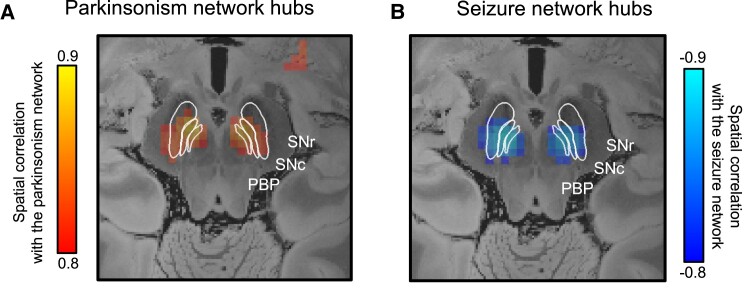
**Parkinsonism and seizure network hubs.** We identified the hubs in the parkinsonism and seizure networks by computing the voxels whose whole-brain connectivity profile was most spatially similar to either the parkinsonism network (Fig. 2A) or the seizure network (Fig. 2B). Voxels in the substantia nigra were most positively correlated to the parkinsonism network (**A**) and most negatively correlated to the seizure network (**B**). SNr, substantia nigra pars reticulata; SNc, substantia nigra pars compacta; PBP, parabrachial pigmented nucleus.

Brain networks for parkinsonism^7,11^ and seizures^8^ were published previously and are shown in Fig. 2A and B. To avoid bias, we first compared the spatial similarity between the published parkinsonism (Fig. 2A) and seizure (Fig. 2B) networks using a spatial correlation (Pearson’s *r*). These networks were highlighted as the primary network in the previous publications but were derived differently. The parkinsonism map represents the mean connectivity profile of 29 lesions causing parkinsonism (Fig. 2A; see Supplementary Fig. 5 from Siddiqi *et al.*^11^). The seizure map represents the mean connectivity profile to subcortical nodes more functionally connected to 347 lesions causing epilepsy versus 1126 lesions not causing epilepsy (Fig. 2B; see Fig. 4A from Schaper *et al.*^8^). Second, to account for connections that may not be specific to parkinsonism or epilepsy, we also compared lesions causing either symptom to the control lesions from the original papers that were not associated with parkinsonism (*n* = 135 strokes causing other non-specific symptoms such as hemiparesis) or seizures (*n* = 1126 strokes, traumas, tumours and tubers without seizures) using a voxel-wise two-sample *t*-test. This voxel-wise comparison generates whole-brain specificity maps derived in the exact same manner. We then compared these group-level whole-brain specificity maps using a spatial correlation (Fig. 2C and D). Third, we compared the network of each individual parkinsonism and seizure lesion to each other (Fig. 2E and F). Specifically, we assessed (i) the whole-brain connectivity profile of each parkinsonism (*n* = 29) and control (*n* = 135) lesion to each seizure lesion (*n* = 347), averaging across all seizure lesions, and (ii) the whole-brain connectivity profile of each seizure (*n* = 347) and control (*n* = 1126) lesion to each parkinsonism lesion (*n* = 29), averaging across all parkinsonism lesions. Finally, as performed in prior studies,^12^ we used the human connectome to identify voxels whose whole-brain connectivity profile was most similar to the published parkinsonism or seizure network (‘network hubs’; Fig. 3A and B).

### Statistical analysis

Statistical analyses were performed in MATLAB. Whole-brain lesion network maps were compared using a spatial correlation (Pearson’s *r*), and average spatial correlations were compared between groups using a *t*-test. A two-sided *P* < 0.05 was considered significant, and we corrected for multiple testing using false discovery rate correction.^13^

## Results

### Systematic literature review

We screened 1196 unique records in a systematic literature search (Fig. 1) and identified 18 relevant studies that reported on the relationship between parkinsonism and seizures (Table 1). Of these studies, seven supported qualitative evidence for a positive relationship between the two symptoms, seven a negative, one suggested no relationship and three provided evidence for both a positive and negative relationship.

### Parkinsonism versus seizure network

Lesions causing parkinsonism are connected to a brain network (Fig. 2A) with the opposite spatial topography of lesions causing seizures (Fig. 2B; spatial *r* = −0.85). A similar inverse relationship (spatial *r* = −0.51) was found when computing the connections specific for lesions causing parkinsonism (Fig. 2C) or seizures (Fig. 2D). On an individual lesion level, the connectivity profile of lesions causing seizures showed a negative spatial correlation to the connectivity profile of lesions causing parkinsonism {average spatial *r* [95% confidence interval (CI)] = −0.042 (−0.0484:−0.0360); one-sample *t*-test *P* < 0.001; Fig. 2E and F}, and this negative spatial correlation was stronger compared to control lesions not associated with parkinsonism [average spatial *r* (95% CI) = −0.004 (−0.035:0.028); two-sample *t*-test *P* = 0.018]. Vice versa, parkinsonism lesions were more negatively correlated with seizure lesions than control lesions not associated with seizures [average spatial *r* (95% CI) = 0.213 (0.183:0.242); two-sample *t*-test *P* < 0.001]. On a brain network level, lesions causing parkinsonism are thus inversely related to lesions causing seizures.

Finally, we found that the network hubs (voxels with strongest positive or negative spatial correlation) in the published parkinsonism (Fig. 2A) and seizure (Fig. 2B) networks were in the substantia nigra for both symptoms. However, the sign of the association was inverted. Voxels in the substantia nigra were most positively correlated to the parkinsonism network (Fig. 3A) and most negatively correlated (‘anti-correlated’)^14,15^ to the seizure network (Fig. 3B).

## Discussion

In this study, we found that brain lesions causing parkinsonism and lesions causing seizures map to opposite brain networks, providing neuroanatomical insight into conflicting epidemiological evidence.^1,4^

Our results using brain lesions causing parkinsonism or seizures offer a unique perspective and suggest that parkinsonism and epilepsy are inversely related on a brain network level. This inverse relationship is consistent with multiple clinical and epidemiological findings of an inverse relationship, including reports of seizures improving parkinsonism^4,5^ and the onset or progression of parkinsonism improving seizures^1,4^ (see Table 1). Our results may also aid in interpreting prior epidemiological studies that observed a positive association.^2,3^ Specifically, our results are consistent with the hypothesis that treatment of one disorder (not the presence of the disorder itself) may increase the risk of the other disorder, resulting in a positive association. Examples include anti-seizure drug–associated tremor and Parkinson’s disease.^3,6^ While it is unknown whether our results generalize to idiopathic parkinsonism or epilepsy not caused by lesions, mapping of lesions causing new-onset symptoms can allow for causal links between neuroanatomy and symptoms,^16^ akin to how Mendelian randomization allows for causal links between genes and symptoms.^17^

Regarding neuroanatomy, we found that the substantia nigra was a network hub in both the parkinsonism and seizure networks, but the sign of the association was reversed. This means that the substantia nigra is the most likely location in the brain where a lesion would be expected to cause parkinsonism, consistent with the pathophysiology of Parkinson’s disease. However, it is the least likely location for a lesion to cause seizures and could potentially improve or protect against seizures. This result is consistent with Yakovlev’s^1^ clinical observations^4^ and results in experimental animals where direct lesioning, high-frequency stimulation or optogenetic inhibition of the substantia nigra reduces seizures.^18^ Future prospective high-resolution neuroimaging studies in humans may investigate regional differences in connectivity of the substantia nigra and its subregions in patients with parkinsonism or epilepsy such as those previously identified in animal models.^18-20^

The finding of opposing brain networks for parkinsonism and epilepsy may offer one possible explanation ‘why’ seizures can disappear with the onset of parkinsonism,^1,4^ ‘why’ seizures can improve parkinsonism^4,5^ and ‘why’ anti-seizure drugs may increase the risk of parkinsonism.^6^ Although speculative, one disease may compensate for the other, in line with Yakovlev’s^1^ hypothesis from 1928. Finally, these results may also have implications for side effect monitoring during the development of new treatments. Treatments targeting the brain network of one disease could worsen the other. For example, epidemiological data suggest an increased risk of parkinsonism with common anti-seizure drugs.^6^ Conversely, D_1_ selective medications are being explored as a more effective option for parkinsonism, but experimental data suggests these medications may confer a higher risk for seizures.^21^ Future studies using pharmaco-MRI may be able to prospectively test these hypotheses in humans and measure the brain network states associated with potential side effects of new treatments.^22^

## Data Availability

This paper used de-identified data from different teams of investigators at various institutions, across different countries. Each data set is available upon reasonable request from each respective team of investigators. Data sharing will be subject to the policies and procedures of the institution as well as the laws of the country where each data set was collected.

## References

[1] Yakovlev PI . Epilepsy and parkinsonism. N Engl J Med. 1928;198(12):629–638.

[2] Gruntz K , Bloechliger M, Becker C, et al Parkinson disease and the risk of epileptic seizures. Ann Neurol. 2018;83(2):363–374. 29369409 10.1002/ana.25157

[3] Simonet C , Bestwick J, Jitlal M, et al Assessment of risk factors and early presentations of Parkinson disease in primary care in a diverse UK population. JAMA Neurol. 2022;79(4):359–369. 35254398 10.1001/jamaneurol.2022.0003 PMC8902684

[4] Vercueil L . Parkinsonism and epilepsy: Case report and reappraisal of an old question. Epilepsy Behav EB. 2000;1(2):128–130. 10.1006/ebeh.2000.0044 12609142

[5] Takamiya A , Seki M, Kudo S, et al Electroconvulsive therapy for Parkinson’s disease: A systematic review and meta-analysis. Mov Disord. 2021;36(1):50–58. 33280168 10.1002/mds.28335

[6] Belete D , Jacobs BM, Simonet C, et al Association between antiepileptic drugs and incident Parkinson disease. JAMA Neurol. 2023;80(2):183–187. 36574240 10.1001/jamaneurol.2022.4699 PMC9857018

[7] Joutsa J , Horn A, Hsu J, Fox MD. Localizing parkinsonism based on focal brain lesions. Brain J Neurol. 2018;38(Pt 10):S67–S2456. 10.1093/brain/awy161 PMC6061866 29982424

[8] Schaper FLWVJ , Nordberg J, Cohen AL, et al Mapping lesion-related epilepsy to a human brain network. JAMA Neurol. 2023;80(9):891–902. 37399040 10.1001/jamaneurol.2023.1988 PMC10318550

[9] Boes AD , Prasad S, Liu H, et al Network localization of neurological symptoms from focal brain lesions. Brain J Neurol. 2015;138(Pt 10):3061–3075. 10.1093/brain/awv228 PMC4671478 26264514

[10] Fox MD . Mapping symptoms to brain networks with the human connectome. N Engl J Med. 2018;379(23):2237–2245. 30575457 10.1056/NEJMra1706158

[11] Siddiqi SH , Schaper FLWVJ, Horn A, et al Brain stimulation and brain lesions converge on common causal circuits in neuropsychiatric disease. Nat Hum Behav. 2021;5(12):1707–1716. 34239076 10.1038/s41562-021-01161-1 PMC8688172

[12] Joutsa J , Moussawi K, Siddiqi SH, et al Brain lesions disrupting addiction map to a common human brain circuit. Nat Med. 2022;28(6):1249–1255. 35697842 10.1038/s41591-022-01834-y PMC9205767

[13] Benjamini Y , Hochberg Y. Controlling the false discovery rate: A practical and powerful approach to multiple testing. J R Stat Soc Ser B Methodol. 1995;57(1):289–300.

[14] Fox MD , Snyder AZ, Vincent JL, Corbetta M, Van Essen DC, Raichle ME. The human brain is intrinsically organized into dynamic, anticorrelated functional networks. Proc Natl Acad Sci U S A. 2005;102(27):9673–9678. 15976020 10.1073/pnas.0504136102 PMC1157105

[15] Murphy K , Fox MD. Towards a consensus regarding global signal regression for resting state functional connectivity MRI. NeuroImage. 2017;154:169–173. 27888059 10.1016/j.neuroimage.2016.11.052 PMC5489207

[16] Siddiqi SH , Kording KP, Parvizi J, Fox MD. Causal mapping of human brain function. Nat Rev Neurosci. 2022;23(6):361–375. 35444305 10.1038/s41583-022-00583-8 PMC9387758

[17] Sanderson E , Glymour MM, Holmes MV, et al Mendelian randomization. Nat Rev Methods Primer. 2022;2:6. 10.1038/s43586-021-00092-5 PMC7614635 37325194

[18] Wicker E , Beck VC, Kulick-Soper C, et al Descending projections from the substantia nigra pars reticulata differentially control seizures. Proc Natl Acad Sci U S A. 2019;116(52):27084–27094. 31843937 10.1073/pnas.1908176117 PMC6936676

[19] Albala BJ , Moshe´ SL, Cubells JF, Sharpless NS, Makman MH. Unilateral peri-substantia nigra catecholaminergic lesion and amygdala kindling. Brain Res. 1986;370(2):388–392. 3085869 10.1016/0006-8993(86)90500-7

[20] Moshé SL , Garant DS, Sperber EF, Velísková J, Kubová H, Brown LL. Ontogeny and topography of seizure regulation by the substantia nigra. Brain Dev. 1995;17(Suppl 1):61–72. 8882575 10.1016/0387-7604(95)90074-8

[21] Starr MS . The role of dopamine in epilepsy. Synapse. 1996;22(2):159–194. 8787131 10.1002/(SICI)1098-2396(199602)22:2<159::AID-SYN8>3.0.CO;2-C

[22] Xiao F , Koepp MJ, Zhou D. Pharmaco-fMRI: A tool to predict the response to antiepileptic drugs in epilepsy. Front Neurol. 2019;10:1203. 31798524 10.3389/fneur.2019.01203 PMC6863979

